# Optimizing Body Composition During Weight Loss: The Role of Amino Acid Supplementation

**DOI:** 10.3390/nu17122000

**Published:** 2025-06-13

**Authors:** Daniele Cannavaro, Francesco Leva, Alfredo Caturano, Cesare Celeste Berra, Leonilde Bonfrate, Caterina Conte

**Affiliations:** 1 Department of Endocrinology and Metabolic Diseases, IRCCS MultiMedica, 20099 Milan, Italy; daniele.cannavaro@multimedica.it (D.C.); cesare.berra@multimedica.it (C.C.B.); 2 Department of Human Sciences and Promotion of the Quality of Life, San Raffaele Roma University, 00166 Rome, Italy; francesco.leva@uniroma5.it (F.L.); alfredo.caturano@uniroma5.it (A.C.); leonilde.bonfrate@uniroma5.it (L.B.)

**Keywords:** weight loss, lean body mass, sarcopenia, amino acids, BCAA, bariatric surgery, GLP-1, sarcopenic obesity

## Abstract

**Background/Objectives:** Weight loss interventions in individuals with overweight or obesity result in reductions in both fat mass and lean body mass (LBM). While fat loss is the primary therapeutic target, preserving LBM may have favorable health implications. This narrative review evaluates the role of amino acid supplementation, including essential amino acids (EAAs) and branched-chain amino acids (BCAAs), in supporting the preservation of LBM during weight loss induced by lifestyle interventions, pharmacotherapy, or bariatric surgery. **Methods:** This is a narrative review of preclinical and clinical studies examining the effects of amino acid supplementation during calorie restriction on body composition and, when available, functional outcomes. A comprehensive search was conducted in the PubMed, Scopus, and Web of Science databases. **Results:** Evidence suggests that EAA and peptide-based supplementation may help preserve LBM during periods of reduced energy intake, particularly when protein intake from whole foods is limited. Benefits appear more consistent when supplementation is combined with resistance exercise. BCAA supplementation alone has shown variable effects, especially in sedentary individuals or when total protein intake is already sufficient. Anabolic resistance associated with obesity may attenuate the muscle protein synthesis response to dietary amino acids. **Conclusions:** Amino acid supplementation may support the maintenance of LBM during weight loss, particularly under conditions of low protein intake or in conjunction with exercise. Further research is needed to determine the clinical significance of LBM changes and identify optimal supplementation strategies.

## 1. Introduction

Intentional weight loss is an established strategy to improve cardiometabolic risk factors, reduce obesity-related comorbidities, and enhance the overall quality of life in individuals with overweight or obesity [1,2]. However, weight loss typically comprises both fat mass and lean body mass (LBM), the latter of which plays a key role in metabolic health, physical function, and long-term weight maintenance [3,4,5]. Preserving LBM during weight loss is therefore essential to optimize the quality—not just the quantity—of weight loss.

High-protein diets and resistance training are well-established strategies to mitigate LBM loss during energy restriction [6,7,8]. In recent years, amino acid supplementation, particularly with branched-chain amino acids (BCAAs), has been explored as a targeted intervention to support lean mass preservation during an energy deficit. Among them, leucine, in particular, is recognized as a key regulator of muscle protein synthesis (MPS) via the activation of the mTOR pathway [9], and it has been shown to reduce muscle protein breakdown (MPB) [10]. These dual effects may support the maintenance of LBM during periods of calorie restriction. Other amino acid formulations, including essential amino acids (EAAs) and β-hydroxy β-methylbutyrate (HMB), a leucine metabolite, have demonstrated potential in promoting muscle anabolism and preserving LBM, particularly in catabolic states [11]. Despite promising mechanistic data and some supportive clinical studies, the evidence surrounding the effectiveness of amino acid supplementation for LBM preservation remains mixed, with substantial variability depending on the population, intervention type, dosage, and study duration. Furthermore, most available data focus on specific subgroups, such as athletes or elderly individuals, leaving a gap in understanding its role in broader clinical settings, including bariatric surgery, pharmacological interventions, and general lifestyle-based weight loss strategies. This narrative review aims to summarize current evidence on the role of amino acid supplementation, particularly BCAAs, in preserving LBM during weight loss. We examine the underlying physiological mechanisms, preclinical and clinical findings, and implications across lifestyle, surgical, and pharmacological weight loss interventions. By integrating data from diverse interventions, we aim to provide clinicians and researchers with a clearer understanding of whether and how amino acid supplementation can optimize body composition during weight loss.

## 2. Research Methodology

This narrative review was conducted following a systematic approach involving a literature search, the screening of abstracts and full texts, and the synthesis of findings. A comprehensive search was performed in the PubMed, Scopus, and Web of Science databases up to April 2025 to identify relevant preclinical and clinical studies. Keywords used included combinations of terms such as “amino acid supplementation,” “essential amino acids,” “branched-chain amino acids,” “weight loss,” “calorie restriction,” “body composition,” “lean body mass,” and “muscle preservation.” After removing duplicates, abstracts were screened to ensure alignment with the review objectives. Studies were included if they examined the effects of amino acid supplementation on body composition, particularly LBM, during weight loss induced by lifestyle interventions, pharmacotherapy, or bariatric surgery. Given the narrative nature of this review, no formal systematic review protocol or meta-analysis was conducted. Eligible studies were summarized and synthesized to provide an integrated overview of current evidence and identify gaps for future research.

## 3. Mechanisms: How Amino Acids May Preserve Muscle During Calorie Deficit

**Figure 1:**
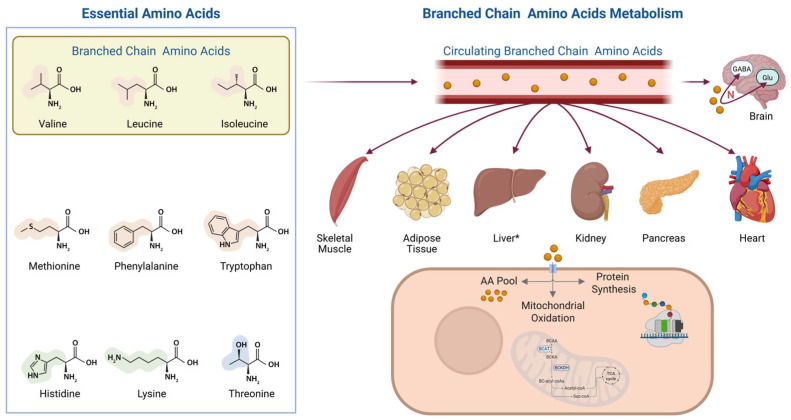
EAAs (**left**) and the metabolism of BCAAs (**right**). BCAAs are taken up and metabolized in extra-hepatic organs, largely due to the low expression of BCAT in hepatocytes. * Some degree of BCAA transamination may occur in the liver through BCAT activity in non-parenchymal hepatic cells. The major sites of BCAA catabolism include skeletal muscle, adipose tissue, the heart, the kidney, and the pancreas. Within these tissues, BCAAs may be directed toward protein synthesis, mitochondrial oxidation, or storage within the intracellular amino acid pool. In the central nervous system, BCAAs serve as nitrogen donors for the synthesis of neurotransmitters such as GABA and glutamate, and they may also act as precursors in sterol and glucose biosynthetic pathways. BCAA oxidation is initiated by a reversible transamination step catalyzed by BCAT1/2, which converts BCAAs into their corresponding BCKAs. These intermediates are then irreversibly decarboxylated by the mitochondrial BCKDH complex, yielding acyl-CoA derivatives. The resulting acyl-CoAs are further metabolized to form acetyl-CoA and succinyl-CoA, which feed into the TCA cycle. BCAAs, branched-chain amino acids; BCAT, branched-chain amino acid transaminase; BCKAs, branched-chain α-keto acids; BKDH, branched-chain α-keto acid dehydrogenase; EAAs, essential amino acids; GABA, γ-aminobutyric acid; Glu, glutamate; N, nitrogen; TCA, tricarboxylic acid. Created in BioRender. Conte, C. (2025) https://BioRender.com/0r3jn43.

EAAs, including histidine, isoleucine, leucine, lysine, methionine, phenylalanine, threonine, tryptophan, and valine, cannot be synthesized de novo by the human body and therefore must be obtained through the diet. Among EAAs, the BCAAs (leucine, isoleucine, and valine) are distinguished by their unique structural and metabolic characteristics (Figure 1).

### 3.1. Metabolic Fate and Oxidation

Unlike most amino acids, BCAAs are primarily catabolized in extrahepatic tissues, particularly skeletal muscle, due to the low hepatic expression of branched-chain aminotransferase (Figure 1) [12,13]. They serve not only as substrates for protein synthesis but also as key regulators of metabolic signaling, as well as sources of nitrogen and carbon for energy production, influencing glucose and lipid metabolism [13].

### 3.2. Anabolic Signaling via mTORC1

Some of these amino acids, especially leucine, have been proposed to play a role in the preservation of lean body mass during energy restriction. Their effects are thought to arise through both anabolic signaling and substrate provision for skeletal muscle metabolism. Leucine is a key regulator of MPS, acting as a potent activator of the mechanistic target of rapamycin complex 1 (mTORC1), a central hub in anabolic signaling. The activation of mTORC1 initiates translation and stimulates MPS, even under conditions of limited energy availability [14,15,16,17], such as when the basal rates of protein synthesis are typically reduced. Of note, recent evidence suggests that the mTORC1 pathway is downregulated during therapy with glucagon-like peptide 1 (GLP-1) receptor agonist [18], although the clinical relevance of this finding is unclear.

### 3.3. Anti-Catabolic Mechanisms

In addition to its anabolic role, BCAA supplementation has been shown to reduce protein breakdown. Experimental studies indicate that BCAAs can downregulate the expression of muscle-specific ubiquitin ligases such as muscle atrophy F-box (MAFbx/atrogin-1) and muscle RING-finger protein-1 (MuRF-1), both of which are involved in the ubiquitin–proteasome degradation pathway [19]. By reducing the expression of these catabolic markers, BCAAs may help preserve muscle mass during periods of negative energy balance.

### 3.4. Energy Substrate Role

Unlike most amino acids, which are primarily catabolized in the liver, BCAAs are metabolized directly within skeletal muscle and other tissues [20,21], where they can serve as an immediate substrate for energy production via oxidization in the tricarboxylic acid cycle [22] and increase MPS [23]. During a calorie deficit or physical exertion, this feature may enable BCAAs to function as an alternative energy source, thereby reducing the need for endogenous MPB to support energy demands [24].

### 3.5. Hormonal and Appetite-Related Effects

BCAA supplementation may also exert potential effects on anabolic hormone release. Leucine has been shown to stimulate insulin secretion and increase circulating insulin-like growth factor 1 (IGF-1), both of which support muscle protein accretion [25,26]. However, these findings are primarily derived from experimental studies, and clinical evidence remains limited and inconsistent. Furthermore, evidence suggests that BCAAs may influence appetite-regulating hormones such as leptin [27], potentially contributing to improved fat loss and the preservation of lean mass, although these mechanisms remain speculative and require further validation in human trials.

## 4. Evidence from Preclinical Trials

Preclinical studies provide insights into the potential role of amino acid supplementation in modulating body composition and metabolic health (Table 1). In short-term caloric restriction models, leucine supplementation in calorie-restricted rodents (0.59% added to feed) did not prevent the loss of body fat or lean mass over one week. However, an increase in liver protein content, a potential marker of visceral protein preservation, suggested a modest anti-catabolic effect in non-muscle tissues [28]. These findings indicate that leucine alone is not sufficient to mitigate rapid muscle loss during acute calorie restriction. By contrast, in chronic catabolic models, such as malnourished aged rats, leucine-enriched refeeding dramatically improved muscle recovery, increasing muscle mass by approximately 51% and doubling muscle strength compared to the controls [29].

Chronic elevations in circulating BCAA levels and/or their immediate catabolic intermediates are associated with obesity and insulin resistance in both animal models and humans [12]. Impaired BCAA catabolism exacerbates metabolic dysfunction, suggesting that context is critical. In energy-surplus states, high BCAA levels may contribute to metabolic inflexibility. However, during energy restriction, targeted BCAA supplementation may selectively support muscle preservation without adverse metabolic effects. For example, while a reduction in circulating BCAAs following weight loss correlates with improved metabolism and reduced hepatic and abdominal fat [30,31], BCAA supplementation during an energy deficit has been shown to help maintain muscle mass [30]. Notably, experimental evidence suggests that the metabolic improvements observed after bariatric surgery in mice models are not necessarily driven by reductions in BCAA levels [30]. Importantly, studies evaluating the impact of BCAA supplementation on insulin sensitivity yield mixed results. Some studies in mice with diet-induced obesity suggest that BCAA or leucine supplementation may worsen insulin sensitivity [32], while others do not support this association [33,34,35,36]. These inconsistencies may reflect differences in the dosage, diet composition, or obesity model used. In contrast, human studies conducted during weight loss interventions did not find a significant effect of BCAA supplementation on insulin sensitivity [37]. Overall, these findings highlight the context-dependent effects of BCAAs, which are influenced by factors such as baseline metabolic health, age, and energy status.

**Table 1:** Summary of key preclinical studies investigating the role of leucine and BCAAs in metabolism, obesity, and insulin resistance.

	Model	Intervention	Main Findings
Pedrosa et al. [28]	Adult male Wistar rats under 50% food restriction	Leucine supplementation (0.59%)	Increased liver protein content but no effect on body fat
Faure et al. [29]	Aged Sprague Dawley rats with dietary restriction	Leucine or citrulline during refeeding	Improved muscle mass and function; only leucine increased muscle force
Bozadjieva Kramer et al. [30]	Post-vertical sleeve gastrectomy in mice	BCAA levels after bariatric surgery	Lower BCAA levels post-surgery; BCAA decrease not required for improved glucose tolerance
Zhang et al. [32]	Obese and lean mice with/without exercise	BCAA supplementation during running exercise	In obese mice, BCAA supplementation reversed exercise-induced improvements in insulin sensitivity; ↑ lipogenesis and ↓ Akt phosphorylation in adipose tissue
Binder et al. [33]	HFD-fed mice ± leucine	Leucine supplementation for 17 wks	Reduced fat mass, improved insulin sensitivity, and increased UCP-3; no benefit in already obese mice
Huang et al. [34]	HFD-fed mice + preadipocyte culture	BCAA supplementation	Inhibited adipogenesis via the NADPH-FTO-m6A pathway; reduced CDK2 and CCNA2
Li et al. [35]	HFD-fed mice	Leucine supplementation	Prevented obesity, hyperglycemia, and dyslipidemia (details truncated)
Zhang et al. [36]	Mice on chow or HFDs	Leucine in drinking water (↑ intake)	↓ Weight gain (32%), ↓ adiposity (25%), ↓ LDL (53%), ↑ UCP3 expression, ↓ glucose, and ↑ insulin sensitivity in HFD mice

↓—decrease, ↑—increase, BCAAs—Branched-Chain Amino Acids, CCNA2—Cyclin A2, CDK2—Cyclin-Dependent Kinase 2, FTO—Fat Mass and Obesity-Associated Protein, HFD—High-Fat Diet, LDL—Low-Density Lipoprotein, m6A—N6-Methyladenosine, NADPH—Nicotinamide Adenine Dinucleotide Phosphate (Reduced Form), and UCP3—Uncoupling Protein 3.

## 5. Evidence from Clinical Trials

### 5.1. Lifestyle-Induced Weight Loss

A lifestyle intervention remains the first-line therapy for achieving moderate weight loss in individuals with overweight and obesity [38]. Through caloric restriction, increased physical activity, and behavioral modifications, lifestyle changes improve insulin sensitivity primarily by reducing adiposity and lowering systemic inflammation, thereby improving insulin sensitivity [39]. These changes arise from enhanced skeletal muscle glucose uptake via the upregulation of insulin-stimulated GLUT4 translocation and reduce hepatic glucose production, leading to improvements in both peripheral and hepatic insulin sensitivity [40]. At the cellular level, reduced inflammation and lipid accumulation in skeletal muscle and the liver restore insulin signaling pathways, particularly by reducing the serine phosphorylation of IRS-1 and increasing the activation of the PI3K-Akt pathway [41]. Physical activity further enhances mitochondrial biogenesis and oxidative capacity, promoting metabolic flexibility, the capacity to adapt substrate utilization to changes in metabolic state, and improving glucose disposal at the muscular level [42,43]. Additionally, lifestyle interventions may modestly improve pancreatic β-cell function, especially in the early or prediabetic stages, by alleviating glucotoxicity and lipotoxicity [44]. However, improvements in insulin secretion tend to be less pronounced than changes in insulin sensitivity and generally require sustained adherence to lifestyle modifications [44,45].

In parallel, lifestyle interventions exert significant effects on amino acid metabolism [46]. Weight loss and improved insulin sensitivity are typically accompanied by reductions in circulating BCAAs, which are elevated in insulin-resistant states and contribute to impaired glucose metabolism via mTORC1 overactivation and the accumulation of toxic metabolites such as BCKAs in muscle and the liver [6,47]. Physical activity promotes the increased oxidation of BCAAs in skeletal muscle, while dietary modulation can influence amino acid profiles and the nitrogen balance [48]. Furthermore, caloric restriction may reduce the flux through amino acid catabolic pathways in the liver, contributing to systemic metabolic improvements [49]. Notably, dietary composition, especially protein quality and distribution, also influences amino acid metabolism during weight loss [50]. Increasing dietary protein helps preserve lean mass during weight loss in adults with overweight or obesity, with intake levels above 1.3 g/kg/day even being associated with gains in muscle mass [51]. However, higher protein consumption does not appear to significantly preserve muscle strength or physical function [38].

Emerging evidence suggests that when the total dietary protein intake is adequate, additional supplementation with BCAAs may offer limited benefits in preserving LBM during weight loss. In a large 16-week randomized controlled trial involving 132 adults with overweight or obesity, three dietary interventions were compared: a standard-protein diet (14% of total energy intake), the same diet supplemented with BCAAs (~0.1 g/kg body weight), and a high-protein diet (27% of the total energy intake) [37]. While weight loss was comparable across groups (~8% of the initial body weight), modest differences in lean mass loss were observed (4.4% in the BCAA group versus 5.4% in the standard-protein group and 3.7% in the high-protein group). However, these differences did not reach statistical significance, and thus the findings should be interpreted cautiously. This suggests that BCAA supplementation offers limited additive benefits in the context of adequate protein intake. Emerging evidence has explored EAA supplementation in structured dietary programs. A 2024 retrospective study assessed the effect of adding EAAs to a structured weight loss program incorporating a very-low-energy ketogenic diet (VLEKD) [52]. Following a 45-day VLEKD, young women with obesity entered a carbohydrate reintroduction period, during which one group received 8 g/day of EAAs, while the control group received no supplementation. Although both groups achieved comparable weight loss during the VLEKD (~7% of the initial body weight), notable differences emerged during refeeding. Participants receiving EAAs exhibited significantly greater reductions in total body weight and fat mass compared to the controls (*p* < 0.001). Importantly, only the EAA group demonstrated improvements in LBM parameters assessed by bioimpedance analysis—including increased relative muscle mass and body cell mass—and handgrip strength. These findings suggest that targeted EAA supplementation may be effective in supporting muscle retention and optimizing body composition following intensive weight loss. However, given the retrospective design of the study, RCTs are needed to confirm these results.

Amino acids have also been incorporated into meal replacement strategies to optimize body composition during calorie restriction. In a small, 8-week randomized trial, Coker et al. investigated the effects of an EAA-enriched meal replacement (EAAMR) strategy compared to a standard isocaloric formula on body composition and the protein fractional synthetic rate, measured using the tracer methodology, in older adults with obesity following a hypocaloric diet [53]. Despite comparable weight loss (~7% of the initial body weight), the weight loss composition differed significantly between groups. Participants receiving EAAMR experienced a significantly greater reduction in fat mass as compared to the control group. While both groups exhibited modest reductions in LBM, the differences were not statistically significant, indicating that EAA supplementation did not fully prevent LBM loss. Nevertheless, the preferential reduction in fat mass observed in the EAAMR group suggests a muscle-sparing effect potentially mediated by enhanced MPS, which was significantly higher in the EAA group. In a more recent 4-week trial, daily supplementation with EAAMR in older adults with obesity resulted in significant fat loss along with the preservation of LBM [54]. Unlike the isocaloric control group receiving a standard meal replacement diet, the EAA group maintained whole-body LBM and showed a modest but statistically significant increase in the thigh muscle cross-sectional area (+2.4 cm^2^ vs. −1.8 cm^2^ in the controls). Physical function, as assessed by the 6 min walk test, also improved. Notably, these benefits occurred without a concurrent exercise program, suggesting that a balanced EAA formulation can support muscle preservation during calorie restriction. Collectively, these findings indicate that integrating EAAs into hypocaloric diets may shift the weight loss composition in favor of fat over LBM.

Amino acid supplementation may exert greater efficacy in preserving LBM when combined with exercise training, particularly during periods of calorie restriction. A randomized trial involving resistance-trained men adhering to a hypocaloric diet compared the effects of BCAA supplementation versus carbohydrate supplementation, with both groups engaging in structured heavy resistance exercise [55]. After 8 weeks, participants in the BCAA group maintained their LBM, exhibiting no significant muscle loss, whereas the carbohydrate group experienced a reduction of approximately 0.9 kg in LBM. Additionally, the BCAA-supplemented group demonstrated superior reductions in fat mass and greater improvements in muscle strength, as evidenced by greater gains in one-repetition maximum (1-RM) performance for both squat and bench press exercises. These findings suggest that peri-exercise BCAA supplementation may support muscle preservation and enhance performance outcomes during energy restriction. However, it is important to acknowledge that the control group received only carbohydrate supplementation without additional protein, potentially conferring a protein intake advantage to the BCAA group and limiting study conclusions. Another randomized controlled trial in middle-aged men with severe obesity compared 4 weeks of a low-calorie diet in combination with exercise and different supplements: no supplement (control), whey protein, BCAAs, or an EAA mixture (with tricarboxylic acid cycle intermediates) [56]. The groups lost similar body weight, and body composition outcomes were different. Only the EAA-supplemented group showed a significant increase in muscle mass, as assessed by bioimpedance analysis (+2.84 kg, +3.6% over baseline) during the weight loss phase, whereas the control group lost muscle mass (−2.46 kg). The whey protein and BCAA groups did not significantly differ from the control in LBM changes [56]. However, the inclusion of tricarboxylic acid intermediates could have confounded the anabolic response, making it difficult to isolate the effect of EAAs alone.

In summary, most studies suggest that BCAA supplementation alone has a modest impact on muscle preservation when total protein intake is adequate (Table 2). Whole-protein sources such as whey or soy, providing BCAAs alongside all EAAs [57,58], might be preferred to ensure optimal substrate availability and, therefore, superior in supporting muscle protein synthesis and body composition during weight loss.

### 5.2. Bariatric Surgery

Bariatric surgery remains the most effective intervention for achieving and sustaining significant weight loss in individuals with obesity [60]. Beyond marked reductions in adiposity, it leads to rapid improvements in insulin sensitivity and glucose metabolism, often resulting in partial or the complete remission of type 2 diabetes [60]. These metabolic benefits are partly independent of weight loss and are mediated by early hormonal changes, including enhanced secretion of incretin hormones such as GLP-1 and PYY, alterations in bile acid metabolism, changes in gut microbiota composition, and reduced hepatic glucose production [61]. At the cellular level, the improvements in insulin sensitivity are driven by reduced adipose tissue inflammation and lipotoxicity, leading to decreased levels of circulating free fatty acids and lipid intermediates such as ceramides and diacylglycerols [62]. This alleviates the inhibition of insulin signaling pathways in insulin-responsive tissues like muscle and the liver, particularly enhancing the IRS–PI3K–Akt cascade, which improves glucose uptake and glycogen synthesis [63]. Furthermore, early post-surgical increases in circulating GLP-1 enhance cAMP signaling in pancreatic β-cells, augmenting insulin biosynthesis and secretion in response to glucose stimulation [64].

Bariatric surgery also influences amino acid metabolism. Postoperative reductions in BCAAs, which are elevated in insulin-resistant states, have been consistently observed [65]. This decrease is thought to reflect both reduced dietary intake and improved catabolism in peripheral tissues [66]. Because elevated BCAAs are linked to impaired insulin signaling [67], their normalization may contribute to enhanced insulin sensitivity following surgery. However, bariatric surgery, beyond inducing substantial weight loss, also results in lean body mass reduction, with the majority of which occurring within the first few postoperative months [68]. Meeting protein requirements after bariatric surgery often becomes a significant challenge due to the reduction in gastric volume and consequent limitations on food intake, particularly during the first postoperative weeks. Current guidelines recommend adopting a relatively high protein intake during the initial months following surgery to preserve LBM [69], although there is some uncertainty as to whether protein supplementation can effectively prevent LBM loss [70], and the amount required to preserve LBM is still debated [71]. Furthermore, patients are often unable to consume sufficient quantities of protein-rich foods [72,73], increasing the risk of protein malnutrition and the subsequent loss of LBM [74]. Amino acid supplementation offers a practical advantage, as a small-volume amino acid-enriched drink or powder can deliver high-quality amino acids without requiring large food portions [75]. Nevertheless, clinical research on this strategy in the context of bariatric surgery remains limited.

A recent trial investigated whether fortifying a whey protein supplement with additional amino acids and vitamin D could enhance the preservation of muscle mass during the first month following sleeve gastrectomy [76]. In this prospective study, 57 participants were assigned to receive either a standard whey protein supplement (40 g/day; n = 26) or the same supplement enriched with BCAAs and 2000 IU of vitamin D daily (n = 31). Body composition and muscle strength were assessed before and one month after surgery. LBM was assessed using multi-frequency BIA under standardized conditions, while muscle strength was evaluated by handgrip dynamometry using the dominant hand. Individuals receiving the BCAA- and vitamin D-fortified protein experienced significantly less loss of LBM compared to those receiving protein alone. After one month, the protein-only group exhibited an approximate 11.4% reduction in LBM, whereas the BCAA/vitamin D group showed a considerably smaller decline of 4.1%. Similarly, the decrease in muscle strength was attenuated in the BCAA-supplemented group (−3.8%) compared to the protein-only group (−18.5%). Of note, both groups achieved comparable total weight loss. Although vitamin D may have contributed to these benefits given its role in muscle function [11], these results suggest that, during the immediate postoperative period when nutritional intake is limited and weight loss is substantial [77], protein supplementation with additional BCAAs can help preserve muscle mass and function. However, as the intervention combined BCAAs and vitamin D, the individual contribution of each component remains unclear. In a prospective study that assessed the effect of three protein supplementation strategies—complex protein (CP), HMB-enriched, and short peptide-based formulas—on the early changes in LBM following Roux-en-Y gastric bypass, after one month the short peptide group had significantly lower LBM loss relative to total weight loss (19.1%) compared to the CP (40.6%) and HMB groups (34.6%), despite experiencing the greatest overall weight reduction [57]. These findings suggest that short peptide-based protein supplementation more effectively preserves lean mass during the early postoperative phase of bariatric surgery (Table 3). However, when looking at the broader body of evidence, a recent meta-analysis including ten randomized controlled trials assessed the effect of protein or amino acid supplementation on anthropometric and body composition outcomes after metabolic bariatric surgery [78]. The results showed modest but statistically significant improvements in weight reduction (weighted mean difference [WMD]: −1.31 kg), muscle mass (WMD: +1.33 kg), and fat-free mass (WMD: +1.74 kg), and fat mass reduction (WMD: −3.91 kg) in the supplemented groups compared to the controls. However, no significant changes were observed in their BMI or overall lean body mass, and the certainty of evidence was rated as moderate to low. These findings suggest that while protein supplementation may confer some benefits to body composition in the postoperative setting, its impact on preserving lean body mass remains uncertain, and further high-quality research is needed to guide clinical recommendations.

### 5.3. Pharmacological Weight Loss

Incretin-based obesity management medications (OMMs) such as GLP-1 receptor agonists (GLP-1 RAs) and tirzepatide promote weight loss primarily through appetite suppression and delayed gastric emptying, leading to reduced caloric intake and subsequent adiposity reduction [80]. Incretin-based agents have been shown to reduce systemic inflammation [81,82] and fat infiltration in the liver and skeletal muscle [83,84,85,86] in different patient populations. At the cellular level, improved insulin sensitivity results from decreased chronic low-grade inflammation and reduced ectopic lipid accumulation in insulin-sensitive tissues such as skeletal muscle and the liver [87]. This reduction in lipid intermediates, including diacylglycerols and ceramides, alleviates lipid-induced insulin resistance by enhancing insulin receptor signaling pathways, such as the insulin receptor substrate (IRS) and phosphatidylinositol 3-kinase (PI3K)/Akt cascade, ultimately improving glucose uptake and metabolism [80,87,88]. Furthermore, GLP-1 RAs and tirzepatide directly enhance glucose-dependent insulin secretion by increasing cyclic AMP (cAMP) levels in pancreatic β-cells, promoting insulin granule exocytosis and β-cell survival. These agents also suppress α-cell glucagon secretion, which reduces hepatic glucose production, contributing to improved glycemic control [80,87,88]. Importantly, these pharmacological interventions also influence amino acid metabolism in the context of weight loss [89]. Enhanced insulin sensitivity facilitates greater amino acid uptake and stimulates protein synthesis in skeletal muscle through the activation of the mTORC1 pathway, which governs muscle protein synthesis and maintenance [90].

Recent studies have demonstrated that weight loss achieved through incretin-based OMMs, such as semaglutide and tirzepatide, is associated with a variable but significant reduction in LBM, typically accounting for approximately 20–40% of the total weight lost [91,92,93]. While some concern exists regarding potential skeletal muscle mass loss, current evidence suggests that the majority of FFM reduction reflects expected physiological changes during weight loss, including reductions in extracellular fluid and organ size, rather than a substantial loss of functional muscle tissue [3,94]. However, it should be acknowledged that direct assessments of muscle tissue loss (e.g., via advanced imaging) are limited, and current interpretations largely rely on body composition techniques like DXA or BIA, which do not measure muscle mass [95].

In SURMOUNT-1, tirzepatide led to greater total weight loss compared to the placebo, but the proportions of fat mass (74%) and LBM (26%) loss were similar to those with the placebo (75% and 25%) [96]. These ratios were consistent across most subgroups stratified by age, sex, and degree of weight loss. Importantly, physical function appears to be maintained or even improved following weight loss with GLP-1 RAs, as observed in trials such as SURMOUNT-1 and STEP-HFpEF, where enhancements in functional outcomes, as assessed using validated patient-reported outcome instruments, were proportional to the amount of weight loss achieved [97,98]. Furthermore, GLP-1 RAs have been associated with favorable changes in muscle quality, such as reductions in intramuscular fat infiltration [85]. Nonetheless, few trials have comprehensively assessed both body composition and functional outcomes, underscoring the need for future studies employing precise muscle mass and strength measurements to fully characterize the clinical implications of LBM loss during GLP-1 RA-induced weight loss and guide clinical practice [95]. Hormonal and metabolic changes during incretin-based therapy, such as decreased insulin and IGF-1 levels, may blunt anabolic signaling pathways like mTOR [99]. Moreover, decreased food intake induced by these agents might lead to reductions in circulating amino acids, possibly impacting MPS. In this context, targeted supplementation with EAAs, particularly leucine-enriched formulations, may help stimulate MPS independently of insulin and support lean mass preservation [100].

Similar to bariatric surgery, the substantial weight loss induced by incretin-based OMMs necessitates careful attention to maintaining adequate protein intake and engaging in regular physical activity. In clinical practice, there is increasing emphasis on promoting high-protein diets for patients undergoing pharmacotherapy with these agents, paralleling guidance typically provided to bariatric surgery patients. Experts recommend a protein intake of approximately 1.5 g per kilogram of ideal body weight for individuals with obesity receiving pharmacologic treatment, particularly for older adults or those who are physically active [69]. This target aligns closely with protein recommendations following bariatric surgery, reinforcing the principle that protein requirements are largely determined by the magnitude of weight loss rather than the method employed. However, the appetite-suppressing effects of GLP-1 RAs may hinder adequate intake not only of protein but also of key micronutrients, such as iron, vitamin B12, and fat-soluble vitamins, especially if dietary variety is reduced [101]. These potential micronutrient deficiencies should be monitored, particularly in long-term therapy, to support muscle protein turnover and preserve LBM.

Currently, there is limited direct research evaluating the effects of BCAA supplementation specifically in patients undergoing incretin-based OMMs. Therefore, the suggestion to integrate amino acid supplementation is speculative and based on extrapolation from studies in lifestyle and bariatric surgery settings. Preliminary evidence suggests that combining GLP-1 RAs with amino acid supplementation and resistance training may enhance the preservation of muscle mass and function [102,103]. Although robust randomized trials are lacking, these findings support the rationale for integrating BCAA or EAA supplements, particularly around exercise or in the context of suboptimal dietary intake, as an adjunct strategy to counterbalance the catabolic effects of GLP-1 RA therapy. In cases where patients experience difficulties in achieving sufficient protein intake due to the appetite-suppressing effects of GLP-1 RAs, supplementation with a BCAA or EAA beverage may help reach the anabolic threshold necessary for muscle maintenance (e.g., leucine intake of approximately 2.5–3 g per meal) [104]. In general, resistance training and the sufficient intake of high-quality, EAA-rich protein sources remain foundational strategies for maintaining LBM during weight loss. When these strategies are in place, additional BCAA supplementation may offer marginal benefits, potentially serving as a low-calorie means to enhance amino acid availability, particularly around exercise sessions or between meals, although further research is warranted.

## 6. Discussion

**Figure 2:**
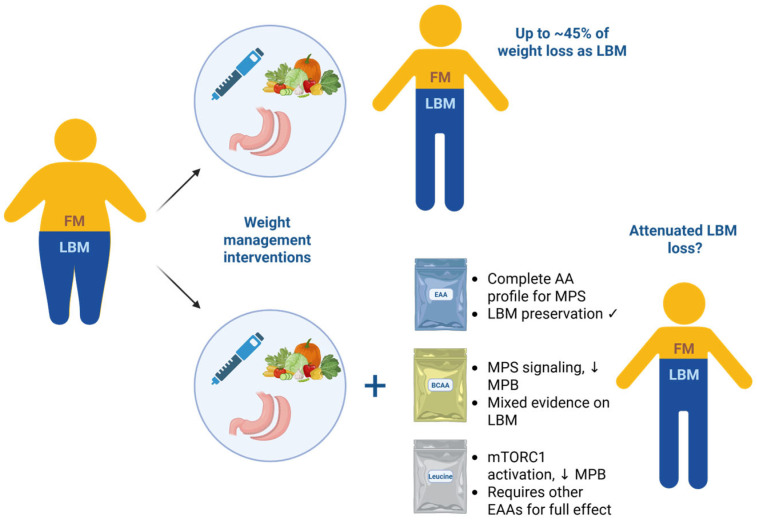
Schematic representation of the changes in body composition during weight loss with weight management interventions (incretin-based obesity management medications, bariatric surgery, and diet) with or without amino acid supplementation. ↓, decrease, ✓ evidence-based, AA, amino acid; BCAAs, branched-chain amino acids; EAAs, essential amino acids; FM, fat mass; LBM, lean body mass; MPB, muscle protein breakdown; MPS, muscle protein synthesis; mTORC1, mammalian target of rapamycin complex 1. Created in BioRender. Conte, C. (2025) https://BioRender.com/z2sf88g.

Significant weight loss is typically accompanied by a reduction in LBM, which may account for up to ~45% of the total weight lost, although the proportion of LBM lost is generally smaller than the reduction in fat mass [4,94,105] (Figure 2). However, the extent of LBM loss may vary depending on the degree of energy restriction and the ability to meet protein requirements. Substantial calorie restriction, as seen after bariatric surgery or during GLP-1 RA therapy, poses a challenge to meeting protein needs for LBM preservation. The degree of calorie restriction directly influences the risk of muscle loss during weight reduction, and the potential utility of amino acid supplementation increases as dietary intake becomes more limited. In the context of moderate energy deficits (e.g., a 500 kcal/day reduction), a high-protein diet alone may be sufficient to preserve lean mass [37]. However, following bariatric surgery (and possibly during incretin-based therapy), particularly in the early postoperative period when the total energy intake may fall below 800 kcal/day, meeting protein requirements through whole foods becomes highly challenging. In such settings, supplemental free-form amino acids (e.g., BCAAs and EAAs) may play a critical role in attenuating muscle loss. EAA formulations and protein hydrolysates (composed of di- and tri-peptides) require minimal digestion and are rapidly absorbed, thereby delivering amino acids efficiently even when gastrointestinal function is compromised. While both BCAAs and EAAs have been used to support skeletal muscle maintenance, EAAs are more likely to support net MPS, particularly when total protein intake is limited. In contrast, BCAAs alone (particularly leucine) may primarily act as signaling molecules to initiate MPS but are insufficient without the full complement of EAAs to sustain it [106,107] (Figure 2). Clinical evidence has shown that supplementation with a short-peptide formula or BCAA plus vitamin D during this period offers additional LBM-preserving benefits beyond protein alone [76,79].

Obesity is increasingly recognized to be associated with a state of anabolic resistance in skeletal muscle, similar to that seen in aging. Individuals with overweight/obesity often exhibit blunted MPS responses to nutrient intake and exercise [108,109], with potential implications for preserving LBM during weight loss. The mechanisms underlying this resistance likely involve chronic low-grade inflammation, insulin resistance, and lipid accumulation in muscle [110]. Furthermore, alterations in the regulation of MPB have also been observed [109], suggesting a complex dysregulation of protein metabolism in obesity. Although not all studies found uniform deficits in muscle anabolic potential [111], most support at least partial anabolic resistance in people with obesity, particularly in response to targeted exercise and protein ingestion, when compared to normal-weight adults [108,109,112,113,114]. Therefore, individuals with overweight or obesity may require higher doses of dietary protein or amino acids, along with more robust exercise stimuli, to elicit anabolic responses comparable to those observed in individuals with a healthy weight.

Resistance exercise is a potent stimulus for MPS, and its interaction with amino acid intake is synergistic. Multiple randomized trials in active individuals have shown that higher protein or amino acid supplementation enhances fat loss and LBM retention when combined with resistance training [71]. When combined with training, supplemental BCAAs may support muscle maintenance and enhance strength adaptations by supplying readily available substrates for muscle protein synthesis [55]. In contrast, it is critical to emphasize that in sedentary individuals, the anabolic stimulus is insufficient to activate MPS, and BCAA supplementation in isolation is unlikely to prevent muscle loss. Thus, the interaction between nutritional support and physical activity is a key determinant of outcomes in LBM preservation during weight loss.

Substantial research gaps remain regarding amino acid supplementation during weight loss. Well-designed randomized controlled trials are needed to directly compare BCAAs, EAAs, and protein hydrolysates in populations such as post-bariatric patients and individuals on incretin-based OMMs. Importantly, studies should assess not only changes in LBM but also functional outcomes, including muscle strength and physical function, to better evaluate clinical efficacy. This may be particularly relevant for older adults and those at risk of sarcopenic obesity [115], where muscle function is a primary concern. An additional aspect that deserves further exploration is the interplay between the absorption of EAAs, particularly BCAAs, and incretin-based OMMs. These medications have well-documented effects on gastrointestinal motility, including delayed gastric emptying and slowed intestinal transit time, which could influence the kinetics of nutrient absorption, including amino acids [116,117,118]. While data on the effects of incretin-based medications on amino acid absorption are not available, studies on the pharmacokinetic effects of GLP-1 receptor agonists consistently show that, although delayed gastric emptying reduces peak concentrations (Cmax) and prolongs the time to peak (Tmax) of oral agents, overall systemic exposure (AUC) remains largely unchanged [119]. These effects are generally not considered clinically significant. In both animal and non-human primate models, dulaglutide did not alter plasma amino acid concentrations, whereas agents with glucagon receptor activity reduced amino acid levels, likely via increased hepatic amino acid uptake and catabolism [120]. Furthermore, incretin-based medications stimulate glucose-dependent insulin secretion, which in turn promotes amino acid uptake and utilization in insulin-sensitive tissues such as skeletal muscle [121,122]. This could theoretically enhance the anabolic response to amino acid supplementation, especially in catabolic or weight-loss states where muscle preservation is a key therapeutic target.

Long-term studies are needed to determine whether the early preservation of LBM translates into sustained functional or metabolic benefits. First and foremost, well-designed head-to-head RCTs directly comparing BCAAs, EAAs, and protein hydrolysates, particularly in populations undergoing pharmacotherapies that influence nutrient intake, such as GLP-1 receptor agonists or dual incretin agonists, are urgently needed. Additionally, given the potential for altered amino acid kinetics due to delayed gastric emptying in these patients, further research should explore the optimal timing, formulation, and dosing of EAA supplementation to maximize efficacy. Finally, elucidating mechanisms of anabolic resistance in obesity may inform adjunct strategies to enhance the effectiveness of amino acid-based interventions.

As a narrative review, this work is inherently limited by its non-systematic methodology. While care was taken to include relevant and high-quality studies, the absence of a formal search strategy and risk-of-bias assessment may introduce selection bias. Additionally, the heterogeneity of the available studies, regarding intervention types, study populations, durations, and outcome measures, limits the ability to draw definitive conclusions or compare findings across different settings. The variability in amino acid formulations and dosages further complicates interpretation. Finally, this review focuses primarily on body composition and does not extensively address functional endpoints, such as muscle strength or performance, which are equally important in clinical practice.

## 7. Conclusions

Regardless of the weight loss method adopted, rapid reductions in body weight without concurrent resistance training are consistently associated with a loss of LBM. Skeletal muscle does not discern the cause of the energy deficit but responds to anabolic stimuli such as EAAs and resistance exercise. Therefore, integrating these strategies is beneficial across all forms of intentional weight reduction, regardless of whether achieved through a diet, pharmacotherapy, or bariatric surgery. In clinical practice, strength-based exercise and, when appropriate, protein or amino acid supplementation are increasingly recommended to promote preferential fat loss and mitigate muscle loss, particularly in patients receiving incretin-based OMMs. As research evidence is still emerging, current recommendations should remain grounded in established nutritional principles: ensure adequate protein intake, consider EAA or BCAA supplementation if protein intake is suboptimal, and encourage consistent engagement in resistance exercise.

Future research should aim to address current gaps in knowledge, including the optimal composition, timing, and duration of amino acid supplementation during various weight loss modalities. Randomized controlled trials across diverse populations, including older adults, individuals with sarcopenic obesity (evaluating also muscle quality and not just quantity), and patients undergoing pharmacological or surgical weight loss, are needed to develop evidence-based guidelines.

## Figures and Tables

**Table 2:** Clinical evidence on the role of essential and branched-chain amino acids in lifestyle-induced weight loss.

	Model	Intervention	Main Findings
Ooi et al. [59]	RCT; 111 Chinese adults with overweight/obesity	BCAA + standard-protein diets vs. the placebo or high-protein diets	BCAA ↑ postprandial fat oxidation and ↓ carb oxidation; no RMR benefit
Annunziata et al. [52]	Women with grade I obesity after the ketogenic diet	8 g/day of EAAs during the reintroduction phase	EAAs improved body composition (↓ fat mass and ↑ muscle mass), ↑ muscle strength, and ↓ hs-CRP vs. the controls
Coker et al. [53]	Older individuals on caloric restriction	EAAMR vs. competitive meal replacement	Both groups lost ~7% body weight; EAAMR showed greater fat loss and ↑skeletal muscle protein FSR
Coker et al. [54]	Older adults with obesity	EMR vs. Optifast^®^ (once/day for 4 weeks)	EMR reduced fat and intrahepatic lipid, ↑ thigh muscle CSA, and ↑ 6 min walk distance
Dudgeon et al. [55]	Resistance-trained males on hypocaloric diet	BCAA vs. CHO with resistance training (8 weeks)	The BCAA group preserved lean mass and improved strength vs. CHO (which lost lean mass)
Brunani et al. [56]	Men with obesity (<65 years, inpatient)	PD-E07 (EAA + TCA), BCAA, and protein vs. the control	Only PD-E07 led to ↑ muscle mass; other groups lost or did not improve muscle mass

↓—decrease, ↑—increase, BCAAs—Branched-Chain Amino Acids, CHO—Carbohydrate, EAAs—Essential Amino Acids, EAAMR—Essential Amino Acid Meal Replacement, EMR—Essential Amino Acid-Enriched Meal Replacement, hs-CRP—High-sensitivity C-Reactive Protein, PD—Pyruvate Dehydrogenase, RCT—randomized clinical trial, RMR—resting metabolic rate, TCA—Tricarboxylic Acid.

**Table 3:** Clinical evidence on the role of essential and branched-chain amino acids in bariatric surgery.

	Model	Intervention	Main Findings
Afsar et al. [72]	RCT-like design with two parallel groups (BSD vs. BSD + PS)	15 g/day protein supplement post-sleeve gastrectomy plus dietitian support	Protein supplementation with dietitian-guided nutrition led to better maintenance of muscle mass compared to the diet alone. Nutritional intake remained below recommended levels in both groups.
Bertoni et al. [73]	Prospective observational	Assessment of protein intake and the use of supplements post-sleeve gastrectomy at 1 and 3 months	Protein intake from food was inadequate. Supplements improved intake, but it was still below recommended levels. Compliance dropped over time.
Moslehi et al. [74]	Cross-sectional	Analysis of dietary macronutrient quality and quantity 2–4 years after SG	Higher protein and fiber intake associated with greater total weight loss and less fat-free mass loss. Poor quality carbs and high fat linked to worse outcomes.
Schiavo et al. [76]	Prospective cohort	Supplementation with protein + BCAA + vitamin D vs. protein alone after SG	Combined supplementation resulted in greater fat mass loss and lower declines in FFM and muscle strength than protein alone.
Maïmoun et al. [77]	Longitudinal prospective	Gender-stratified body composition analysis pre-and 1-month post-SG	Both fat and lean mass decreased post-SG; patterns varied by gender and anatomical site. Men lost more trunk FM; women had lower limb muscle decline.
Comas Martínez et al. [79]	Prospective interventional	Comparison of short peptide-based, HMB-enriched, and complex protein formulas post-RYGB	SPB group had significantly lower %FFM loss despite higher total weight loss, compared to the CP and HMB groups.

BCAAs—branched-chain amino acids, BMI—body mass index, BSD—post-bariatric surgery diet, BW—body weight, CP—complex protein, FFM—fat-free mass, FM—fat mass, HMB—hydroxy methylbutyrate, LBW—lean body weight, PS—protein supplementation, RCT—randomized clinical trial, RYGB—Roux-en-Y gastric bypass, SG—sleeve gastrectomy, SPB—short peptide-based formula.

## Abbreviations

The following abbreviations are used in this manuscript:

BCAA	Branched-chain amino acid
CP	Complex protein
EAA	Essential amino acid
GLP-1	Glucagon-like peptide 1
GLP-1 RA	Glucagon-like peptide 1 receptor agonist
HMB	β-Hydroxy β-methylbutyrate
LBM	Lean body mass
MAFbx	Muscle atrophy F-box (atrogin-1)
MPB	Muscle protein breakdown
MPS	Muscle protein synthesis
MuRF-1	Muscle RING-finger protein-1
mTORC1	Mechanistic target of rapamycin complex 1
OMM	Obesity management medication
VLEKD	Very-low-energy ketogenic diet
1-RM	One-repetition maximum
